# Interleukins Affect Equine Endometrial Cell Function: Modulatory Action of Ovarian Steroids

**DOI:** 10.1155/2014/208103

**Published:** 2014-02-27

**Authors:** Anna Z. Szóstek, Antonio M. Galvão, Takuo Hojo, Kiyoshi Okuda, Dariusz J. Skarzynski

**Affiliations:** ^1^Department of Reproductive Immunology and Pathology, Institute of Animal Reproduction and Food Research, 10-748 Olsztyn, Poland; ^2^Laboratory of Reproductive Endocrinology Graduate School of Natural Science and Technology, Okayama University, 700-8530 Okayama, Japan

## Abstract

The aim of the present study was to investigate the interaction between ovarian steroids, interleukins and prostaglandins (PG) in equine epithelial and stromal cells in vitro. In Experiment 1, cells were exposed to IL-1**α** (10 ng/mL), IL-1**β** (10 ng/mL) or IL-6 (10 ng/mL) for 24 h and cell proliferation was determined using MTT. In Experiment 2, cells were exposed to progesterone (P_4_; 10^−7^ M); 17-**β** estradiol (E_2_; 10^−9^ M) or P_4_+E_2_ for 24 h and later medium was replaced with a fresh one treated with IL-1**α**, IL-1**β** or IL-6 (10 ng/mL, each) for 24 h. The oxytocin (OT; 10^−7^ M) was used as a positive control. In Experiment 3, cells were exposed to P_4_ (10^−7^ M), E_2_ (10^−9^ M) or P_4_+E_2_ for 24 h and the *IL receptor* mRNAs transcription was determined using Real-time PCR. Prostaglandins concentration was determined using the direct enzyme immunoassay (EIA) method. Our findings reveal a functional linking between ovarian steroids and IL-stimulated PG secretion by equine endometrial cells. This interaction could be one of the mechanisms responsible for endometrial local orchestrating events during the estrous cycle and early pregnancy.

## 1. Introduction

Endometrium is a complex tissue, which consists of different cell types. The overriding purpose of endometrial cyclicity is the preparation for embryo implantation. Interactions between prostaglandins (PG) and ovarian steroids play a crucial role in diverse complex processes in several species. The ovarian steroids affect the morphological and functional state of the endometrium. The 17-*β* estradiol (E_2_) regulates sexual behavior, enhances uterine motility, and promotes secretory activity of the entire reproductive tract. In turn, progesterone (P_4_) affects endometrial secretion, promotes the pregnancy maintenance, and inhibits gonadotropin-releasing hormone (GnRH) secretion and reproductive behavior. Ovarian steroids have also been demonstrated to affect PG during the estrous cycle *in vivo* in the mare [1, 2]. Ovarian steroid-stimulated PG secretion by equine endometrial cells was recently evidenced *in vitro* [3, 4]. Prostaglandins act locally, modulating endometrial biological processes, such as cell proliferation, angiogenesis, embryo implantation, or peripherally on corpus luteum (CL) maintenance and luteolysis [5–10].

Interleukins (ILs) are secreted by numerous immune cells, acting mostly in an auto/paracrine manner. Interleukins such as IL-1*α* and IL-1*β* or IL-6 are known to participate in the regulation of endometrial PG synthesis in many species [11–15]. There are two types of IL agonists (IL-1*α* and IL-1*β*) and both of them determine biological responses via specific receptor. Although there are two types of IL-1 receptors (IL-1RI and IL-1RII), only IL-1RI transduces IL-1 signaling response [16]. Interleukin 6 is a pleiotropic cytokine, which is produced by different immune and nonimmune cell types [17]. Interleukin 6 binds to a low-affinity subunit called gp80 or IL-6R*α* on the surface of target cells, promoting tIL-6/IL-6R alpha complex recruitment of signal-transducing subunits called gp130 or IL-6R*β* [17].

In our study, we hypothesized that there is a functional link between IL, PG, and ovarian steroids, where ovarian steroids modulate PG secretion stimulated by IL. To clarify the interaction of those molecules we investigated: (i) the IL influence on PG production and epithelial and stromal cell proliferation, (ii) the modulatory effect of ovarian steroids on IL-stimulated production of PG, and (iii) the effect of ovarian steroids on *IL receptors* mRNAs transcription.

## 2. Materials and Methods

### 2.1. Animals and Endometrial Tissue Collection

Uteri (*n* = 10) from cyclic mares at the early luteal phase of estrous cycle were collected *postmortem*, from April until the end of July at a local abattoir. The mares were healthy as stated by the official governmental veterinary inspection. The estrous cycle phases were identified based on P_4_ and E_2_ analysis in blood serum and an ovary macroscopic observation [18, 19]. The early luteal phase is characterized by the corpora hemorrhagica presence with a plasma concentration of P_4_  > 1 ng/mL. At the mid luteal phase, the developed CL is associated with follicles 15–20 mm in diameter and P_4_  > 6 ng/mL. At the late luteal phase, a regressing CL is present, together with follicles 30–35 mm in diameter and a concentration of P_4_ from 1–2.5 ng/mL. The follicular phase is characterized by an active CL absence and a follicle with various sizes presence but always >35 mm diameter, with a concentration of P_4_  < 1 ng/mL [18, 19]. Moreover, the phases were differentiated, since serum E_2_ was present in basal concentration in luteal phase (around 2 to 10 pg/mL), but it reaches values above 20 pg/mL in the follicular phase [20]. The entire uterus was collected within 5 min of an animal's death, placed in sterile, incomplete (Ca^2+^ and Mg^2+^ free) Hank's balanced salt solution (HBSS) supplemented with gentamicin (20 *μ*g/mL; Sigma-Aldrich, Madison, USA, #G1272) and 0.1% bovine serum albumin (BSA; Sigma-Aldrich, Madison, USA, #A9056), kept on ice, and transported quickly to laboratory. Small pieces of endometrium from each uterus were placed in buffered 4% paraformaldehyde for histological analysis [18], for further classification according to the scoring system developed by Kenney [21]. Only cells derived from category I endometria of Kenney [21] classification were used in the present study. Animal treatment procedures and tissue collection were approved by the Local Animal Care and Use Committee in Olsztyn, Poland (Agreements No. 51/2011).

### 2.2. Epithelial and Stromal Cells Isolation and Culture

A total of 10 uteri from mares in early luteal phase of the estrous cycle were used. The equine epithelial and stromal cells were isolated following the methodology recently described [22]. Cells were cultured at 38°C in a humidified atmosphere of 5% CO_2_. The culture medium was Dulbecco's modified Eagle's medium/nutrient F-12 Ham (DMEM/Ham's F-12; Sigma-Aldrich, Madison, USA; D8900) supplemented with 10% fetal calf serum (FCS; Sigma-Aldrich, Madison, USA; #C6278) and antibiotic and antimycotic solution (Sigma-Aldrich, Madison, USA; #A5955); it was changed every 2 to 3 days. After reaching 90 to 95% confluence (5 or 7 days of the incubation of stromal or epithelial cells, resp.), cells were trypsinized [22].Further, cells were seeded at a density of 5 × 10^5^ viable cells/mL for epithelial cells and 2 × 10^5^ viable cells/mL of stromal cells in 24 or 96-well plates, regarding the experiment. Both cell types viability were over 90%.The cell culture homogeneity was evaluated using immunofluorescent staining for epithelial and stromal cell specific markers (cytokeratin, vimentin, resp.) as described before [22]. The epithelial and stromal cell homogeneity was around 97%.

### 2.3. Experimental Procedures

#### 2.3.1. Experiment 1: The Effect of Interleukin on Epithelial and Stromal Cell Proliferation

Stromal (*n* = 5) and epithelial (*n* = 5) cells derived from passage I were placed in a 96-well plate. After reaching 50% of confluence, the medium was replaced with fresh DMEM without phenol red supplemented with 0.1% BSA and antibiotics and antimycotic solution. Then, cells were incubated with vehicle or with IL-1*α*, IL-1*β*, or IL-6 (10 ng/mL each). After 24 h, cells proliferation was measured by MTT (3-[4,5-dimethylthiazol-2-yl]-2,5-diphenyltetrazolium bromide) method using TOX-1 Kit (Sigma-Aldrich, Madison, USA, #7H258), according to the manufacturer's instructions.

#### 2.3.2. Experiment 2: The Effect of Ovarian Steroids on Cytokine-Stimulated PG Production by Endometrial Cells

**Figure 1:**
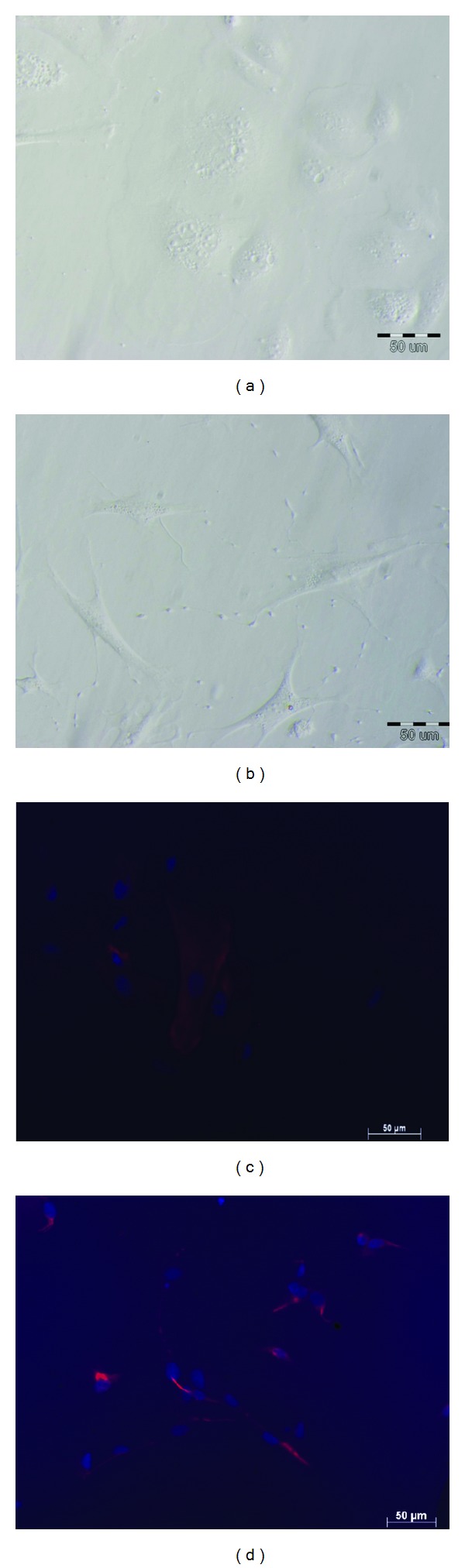
Representative morphology of cultured equine endometrial: (a) epithelial cells and (b) stromal cells. (c) Epithelial cells identification by immunofluorescent staining for cytokeratin; (d) stromal cells identification by immunofluorescent staining for vimentin. The scale bar = 50 *μ*m (magnification: ×40).

Epithelial (Figure 1(a); *n* = 5) and stromal (Figure 1(b); *n* = 5) cells derived from passage I were placed in a 24-well plate in the culture medium DMEM/Ham's F-12 supplemented with 10% FCS and antibiotic and antimycotic solution. Again, when cells reached 90% of confluence, the medium was replaced with fresh DMEM without phenol red (Sigma-Aldrich, Madison, USA; D#2960) supplemented with 0.1% BSA and antibiotics and antimycotic solution. The epithelial and stromal cells were incubated with vehicle or with P_4_ (10^−7^ M), E_2_ (10^−9^ M) or P_4_ + E_2_ (10^−7^ M/10^−9^ M) added to the culture medium for 24 h. The doses for ovarian steroids were determined based on our former work [4]. Further on, the medium was replaced with fresh DMEM without phenol red supplemented with 0.1% BSA and antibiotics and antimycotic solution. Epithelial and stromal cells were then incubated with IL-1*α*/IL-1*β*/IL-6 (10 ng/mL) for the next 24 h. The oxytocin (OT; 10^−7^ M) was used as a positive control. Then, conditioned media were collected into tubes with 5 *μ*L EDTA, 1% acetylsalicylic acid solution (Sigma-Aldrich, Madison, USA, #A2093), and frozen at −20°C until further PG measurement. The total volume of 250 *μ*L TRI Reagent (Sigma-Aldrich, Madison, USA, #T9424) was added to each well containing cells for single-step DNA isolation. Cells from four wells were then polled. Deoxyribonucleic acid was isolated according to TRI Reagent manufacturer procedure. Deoxyribonucleic acid content was used to standardize the results.

#### 2.3.3. Experiment 3: The Effect of Ovarian Steroids on *IL Receptor* mRNAs Transcription in Epithelial and Stromal Cells Culture

Stromal (*n* = 5) and epithelial (*n* = 5) cells derived from passage I were placed in a 24-well plate. When the cells reached 90% of confluence, the medium was replaced with fresh DMEM without phenol red supplemented with 0.1% BSA and antibiotics and antimycotic. Epithelial and stromal cells were incubated with vehicle or with P_4_ (10^−7 ^M), E_2_ (10^−9 ^M) or P_4_ + E_2_ (10^−7 ^M/10^−9 ^M) added to the culture medium for 24 h. After that, culture medium was removed and cells were washed twice with PBS. To each well 250 *μ*L of Fenozol was added and the cells were removed and kept frozen until RNA isolation.

### 2.4. Methods

#### 2.4.1. Total RNA Isolation and cDNA Synthesis

Total RNA was extracted from epithelial and stromal cells from Experiment 2, after culture using the Total RNA Prep Plus Kit (A&A Biotechnology, Gdansk, Poland) according to the manufacturer's instructions. Ribonucleic acid samples were stored at −80°C. Before use, RNA concentration and quality were determined spectrophotometrically and with agarose gel electrophoresis. The absorbance ratio at 260 nm and 280 nm (A_260/280_) was approximately 2. The amount of 1 *μ*g of RNA was reversed transcribed into cDNA using a QuantiTect Reverse Transcription Kit (Qiagen, #205311) according to the manufacturer's instruction. The cDNA was stored at −20°C until real-time PCR was carried out.

#### 2.4.2. Real-Time PCR

Real-time PCR was performed with an ABI Prism 7300 sequence detection system using SYBR Green PCR master mix (Applied Biosystems, Foster City, CA, USA, #4309155). The sequences for equine *IL-1RI*, *IL-1RII*, *IL-6R*β**, and *ACTB* primers were previously published [23]. After a preliminary study, *ACTB* was chosen as the best housekeeping gene. All primers were synthesized by GenoMed (Warszawa, Poland). Total reaction volume was 20 *μ*L and contained 1 *μ*L cDNA (1 ng/*μ*L), 2 *μ*L forward and reverse primers each (250 nM) and 10 *μ*L SYBR Green PCR master mix. Real-time PCR was carried out as follows: initial denaturation (10 min at 95°C), followed by 40 cycles of denaturation (15 s at 95°C) and annealing (1 min at 60°C). After each PCR reaction, melting curves were obtained by stepwise increases in temperature from 60 to 95°C to ensure single product amplification. The product specificity was also confirmed by electrophoresis on 2% agarose gel. The data were analyzed using the method described by Zhao and Fernald [24].

#### 2.4.3. PG and Ovarian Steroids Determination

The concentration of PGE_2_ in the conditioned medium was determined using Prostaglandin E_2_ EIA kit (Cayman) according to the manufacturer's instruction. The concentration of PGF_2*α*_ was determined using the direct enzyme immunoassay (EIA) method as described previously by Uenoyama et al. [25] with modification. The standard curve for PGE_2_ ranged from 16.5 pg/mL to 1000 pg/mL. The intra- and interassay coefficients of variation (CV) were 3.9% and 8.1%, respectively. The standard curve for PGF_2*α*_ ranged from 0.19 ng/mL to 50 ng/mL and CV were 4.7% and 9.8%, respectively.

The concentration of P_4_ in blood plasma was determined using EIA as described previously [26]. The standard curve for P_4_ ranged from 0.0925 ng/mL to 25 ng/mL and intra- and interassay CV were 3.7% and 8.4%, respectively. The antibodies (Anti-P4, code SO/91/4; kindly donated by Dr. S. Okrasa, Warmia-Mazury University, Olsztyn, Poland) were characterized previously [27].

The concentrations of E_2_ in blood serum were assayed by radioimmunoassay (RIA) after extraction with diethyl ether (extraction efficiency: 89%) as described [28]. The antibody (Anti-E2, code BS/88/754; gift from Dr. B. Szafranska, Warmia-Mazury University, Olsztyn, Poland) was characterized previously [29]. The intra- and interassay Cv averaged 4.2% and 8.1%, respectively. The E_2_ standard curve ranged from 0.5 to 80 pg/mL, and the effective dose for 50% inhibition (ED50) of the assay was 1.98 pg/mL. The intra- and interassay Cv averaged 5.2% and 9.5%, respectively.

### 2.5. Statistical Analysis

The data are shown as the mean ± SEM of values obtained in separate experiments, each performed in quadruplicate. The statistical analysis of Experiment 1 and Experiment 3 was performed using parametric one-way ANOVA followed by *Newmann-Keuls* comparison test (GraphPad Software version 5, San Diego, USA). The statistical analysis of Experiment 2 was performed using nonparametric one-way ANOVA *Kruskala*-*Wallisa* followed by *Dunns test*. The results were considered significantly different when *P* < 0.05.

## 3. Results

### 3.1. Experiment 1: The Effect of Ovarian Steroids on Epithelial and Stromal Cell Proliferation

**Figure 2:**
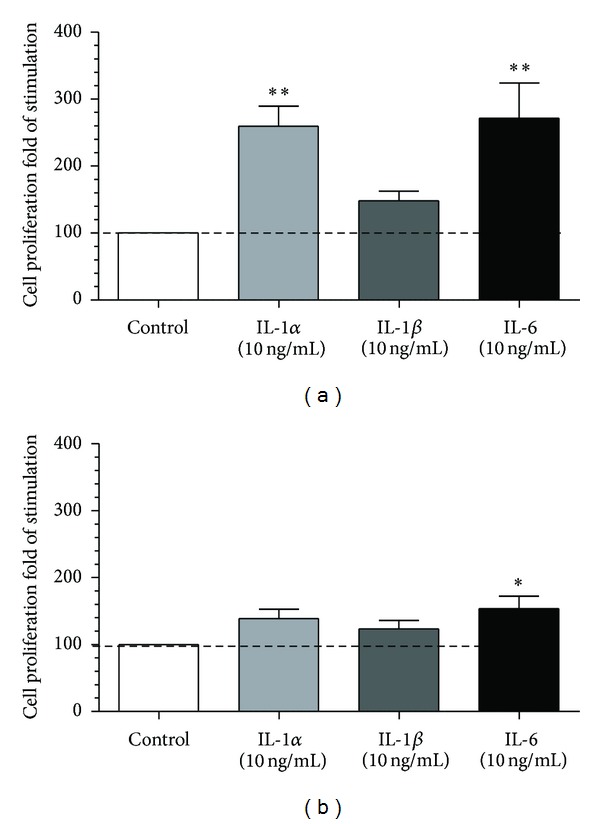
The effect of IL-1*α* (10 ng/mL), IL-1*β* (10 ng/mL), or IL-6 (10 ng/mL) on proliferation of (a) epithelial cells (mean ± SEM; *n* = 5) and (b) stromal cells (mean ± SEM; *n* = 5) after 24 h incubation. All values are expressed as *n-*fold change from control. Asterisks indicate significant differences (*P < 0.05; **P < 0.01) from the respective control, as determined by parametric one-way ANOVA followed by *Newmann-Keuls *comparison test.

Interleukins 1*α* and IL-6 augmented epithelial cell proliferation compared to the control group (Figure 2(a); *P* < 0.01). In turn, only IL-6 augmented stromal cell proliferation compared to the control group (Figure 2(b); *P* < 0.01).

### 3.2. Experiment 2: The Effect of Ovarian Steroids on Cytokine-Stimulated PG Production by Endometrial Cells

**Figure 3:**
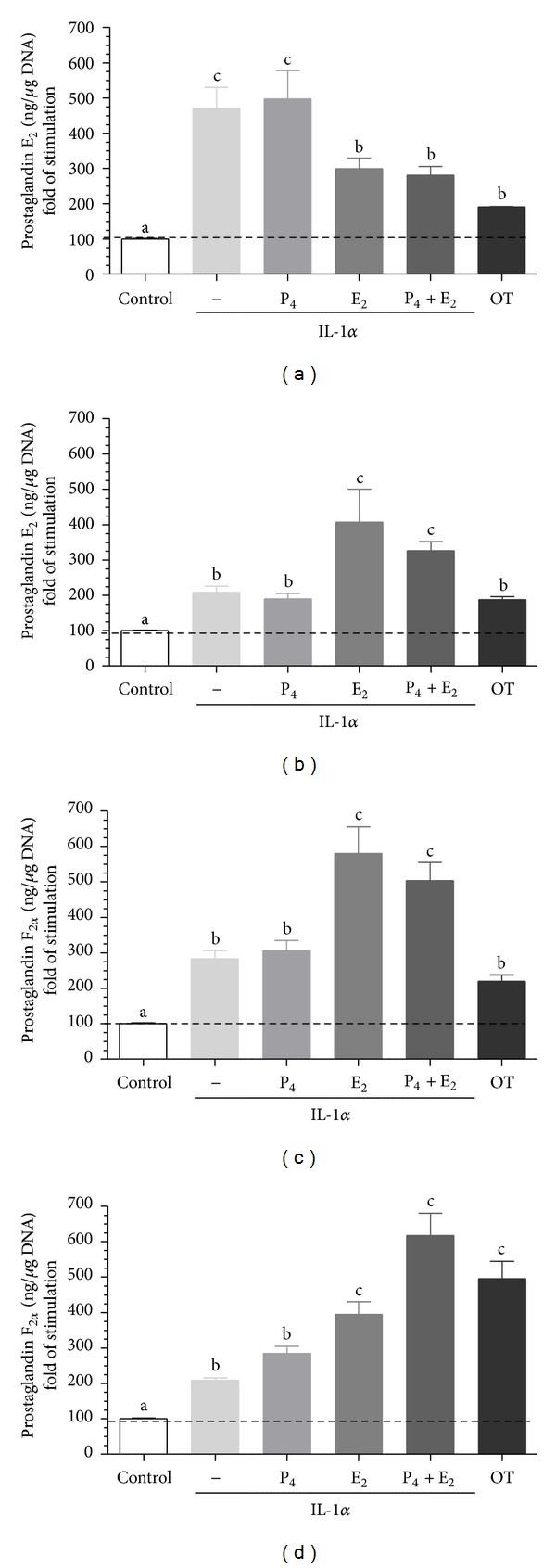
The effect of ovarian steroids on IL-1*α* stimulated PGE_2_ and PGF_2*α*_ production by epithelial ((a), (c)) and stromal ((b), (d)) cells. The cells were treated with P_4_ (10^−7^ M), E_2_ (10^−9^ M), or P_4_ + E_2_ (10^−7^/10^−9^ M) for 24 h. Then, the medium was replaced with a fresh medium and the epithelial cells were stimulated with IL-1*α* (10 ng/mL) for 24 h. Oxytocin (OT; 10^−7^ M) was used as a positive control. All values are expressed as *n-*fold change from control. letters “a,” “b,” and “c” indicate significant differences (*P* < 0.05) between groups, as determined by parametric one-way ANOVA followed by *Newmann-Keuls *comparison test.

The basal *in vitro* PGE_2_ and PGF_2*α*_ secretion from epithelial cells was 1.60 ± 0.924 ng/*μ*g DNA and 2.63 ± 0.343 ng/*μ*g DNA, respectively. The basal *in vitro* PGE_2_ and PGF_2*α*_ secretion from stromal cells was 2.02 ± 0.332 ng/*μ*g DNA and 1.38 ± 0.231 ng/*μ*g DNA, respectively. Oxytocin (positive control) augmented PGE_2_ and PGF_2*α*_ secretion from epithelial cells stromal cells compared to the respective control group (*P* < 0.05; Figure 3 to Figure 5).

Interleukin 1*α* augmented PGE_2_ and PGF_2*α*_ by epithelial (*P* < 0.01; *P* < 0.05; respectively; Figures 3(a) and 3(c)) and stromal cells (*P* < 0.05; Figures 3(b) and 3(d)) compared to the respective control group. Epithelial cells pretreatment with E_2_ or P_4_ + E_2_ decreased IL-1*α*-stimulated PGE_2_ secretion compared to the only IL-1*α*-stimulated group (*P* < 0.05; Figure 3(a)). However, epithelial cells pretreatment with E_2_ or P_4_ + E_2_ augmented IL-1*α*-stimulated PGF_2*α*_ secretion compared to only IL-1*α*-stimulated group (*P* < 0.01; Figure 3(c)). Stromal cells pretreatment with E_2_ or P_4_ + E_2_ augmented IL-1*α*-stimulated PGE_2_ (*P* < 0.001; Figure 6(b)) and PGF_2*α*_ (*P* < 0.001; Figure 3(d)) secretion compared to the respective only IL-1*α*-stimulated group.

**Figure 4:**
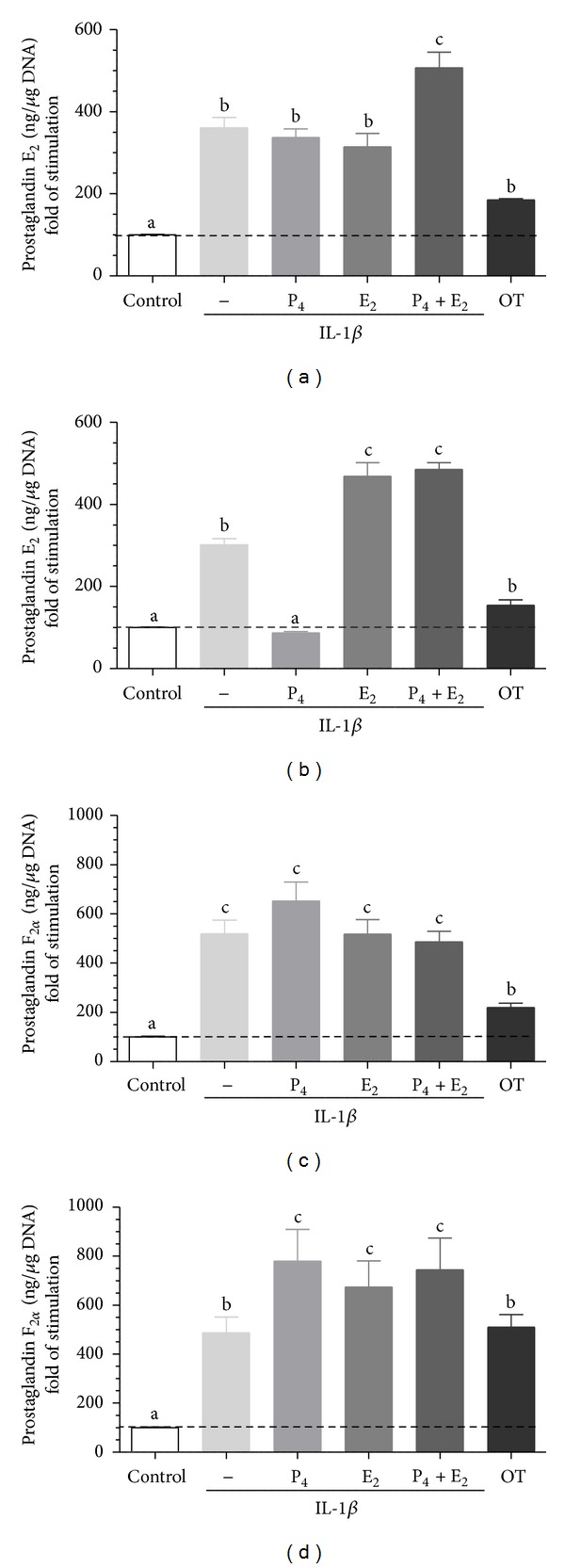
The effect of ovarian steroids on IL-1*β* stimulated PGE_2_ and PGF_2*α*_ production by epithelial ((a), (c)) and stromal ((b), (d)) cells. The cells were treated with P_4_ (10^−7^ M), E_2_ (10^−9^ M), or P_4_ + E_2_ (10^−7^/10^−9^ M) for 24 h. Then, the medium was replaced with a fresh medium and the epithelial cells were stimulated with IL-1*β* (10 ng/mL) for 24 h. Oxytocin (OT; 10^−7^ M) was used as a positive control. All values are expressed as *n-*fold change from control. letters “a,” “b,” “c” indicate significant differences (*P* < 0.05) between groups, as determined by parametric one-way ANOVA followed by *Newmann-Keuls *comparison test.

Interleukin 1*β* augmented PGE_2_ and PGF_2*α*_ by epithelial (*P* < 0.05; *P* < 0.01; respectively, Figures 4(a) and 4(c)) and stromal cells (*P* < 0.05; *P* < 0.01; respectively, Figures 4(b) and 4(d)) compared to the respective control group.

Epithelial cell pretreatment with P_4_ + E_2_ augmented IL-1*β*-stimulated PGE_2_ compared to only IL-1*β*-stimulated group (*P* < 0.01; Figure 4(a)). Stromal cells pretreatment with E_2_ or P_4_ + E_2_ augmented IL-1*β*-stimulated PGE_2_ secretion compared to only IL-1*β*-stimulated group (*P* < 0.01; Figure 4(b)). But stromal cells pretreatment with P_4_ decreased IL-1*β*-stimulated PGE_2_ compared to only IL-1*β*-stimulated group (*P* < 0.05; Figure 4(b)). In turn, stromal cell pretreatment with P_4_, E_2_, or P_4_ + E_2_ augmented IL-1*β*-stimulated PGF_2*α*_ compared to only IL-1*β*-stimulated group (*P* < 0.001; Figure 4(d)).

**Figure 5:**
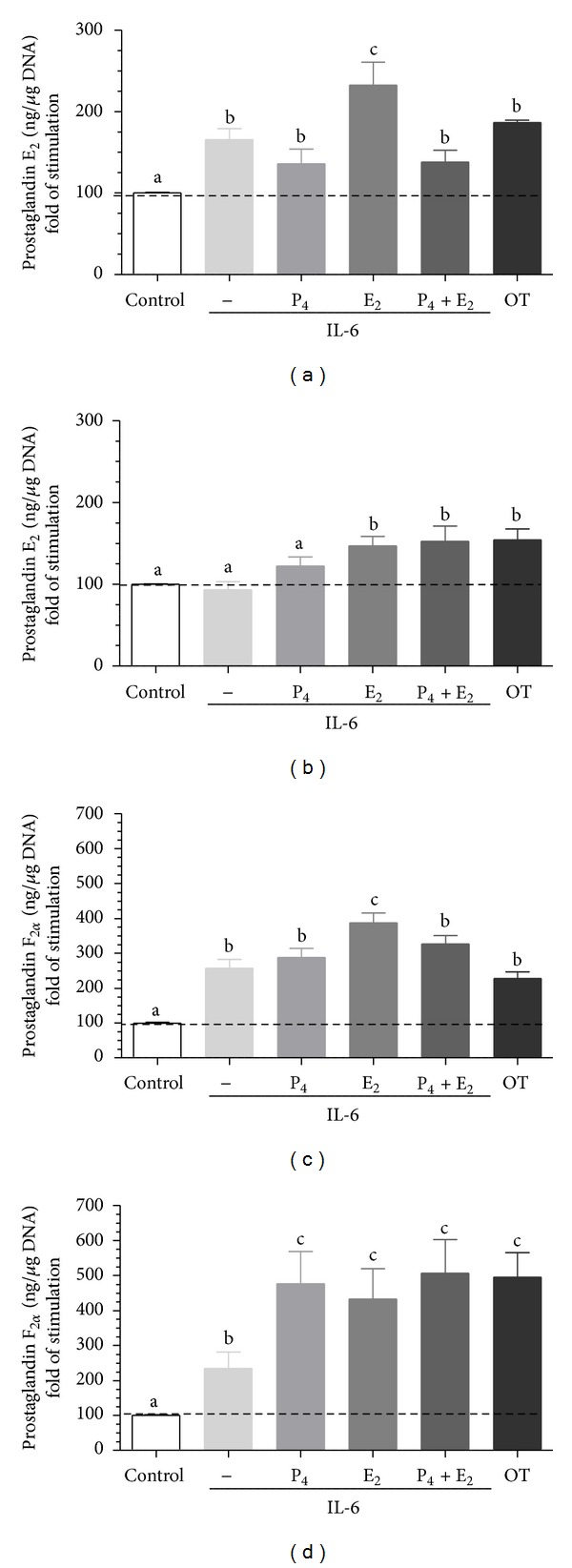
The effect of ovarian steroids on IL-6 stimulated PGE_2_ and PGF_2*α*_ production by epithelial ((a), (c)) and stromal ((b), (d)) cells. The cells were treated with P_4_ (10^−7^ M), E_2_ (10^−9^ M), or P_4_ + E_2_ (10^−7^/10^−9^ M) for 24 h. Then, the medium was replaced with a fresh medium and the epithelial cells were stimulated with IL-6 (10 ng/mL) for 24 h. Oxytocin (OT; 10^−7^ M) was used as a positive control. All values are expressed as *n-*fold change from control. letters “a,” “b,” and “c” indicate significant differences (*P* < 0.05) between groups, as determined by parametric one-way ANOVA followed by *Newmann-Keuls *comparison test.

Interleukin 6 augmented PGE_2_ and PGF_2*α*_ secretion by epithelial cells (*P* < 0.05; Figures 5(a) and 5(c)) and PGF_2*α*_ secretion by stromal cells (*P* < 0.05; *P* < 0.01; respectively, Figure 5(d)) compared to respective control group. Epithelial cell pretreatment with E_2_ augmented IL-6-stimulated PGE_2_ secretion compared to only IL-6-stimulated group (*P* < 0.01; Figure 5(a)). Additionally, stromal cell pretreatment with E_2_ or P_4_ + E_2_ augmented IL-6-stimulated PGE_2_ secretion compared to only IL-6-stimulated group (*P* < 0.01; Figure 5(b)). Stromal cell pretreatment with P_4_, E_2_ or P_4_ + E_2_ augmented IL-6-stimulated PGF_2*α*_ secretion compared to only IL-6-stimulated group (*P* < 0.01; Figure 5(d)).

### 3.3. Experiment 3: The Effect of Ovarian Steroids on *IL Receptor* mRNAs Transcription In Epithelial and Stromal Cells Culture

**Figure 6:**
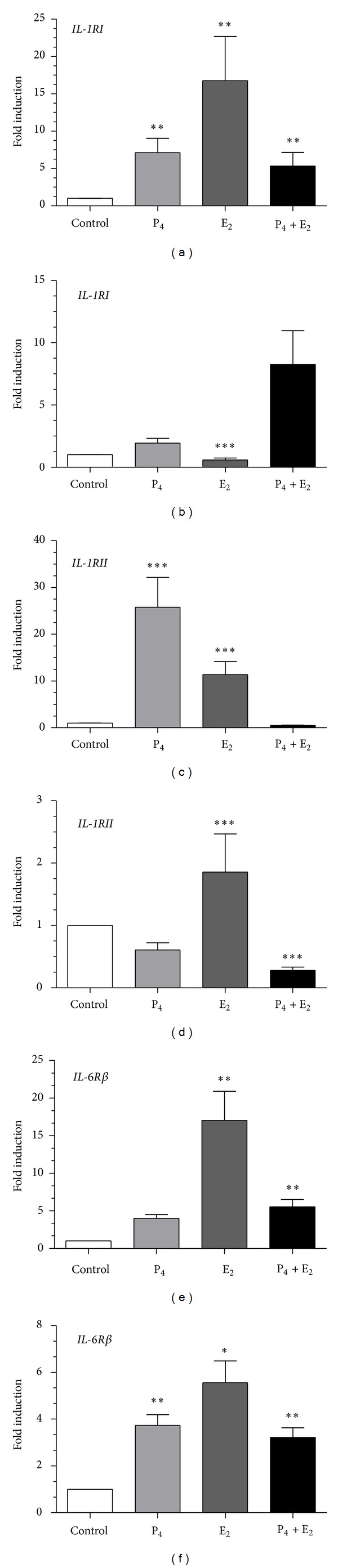
The effect of P_4_ (10^−7^ M), E_2_ (10^−9^ M), and P_4_ + E_2_ (10^−7^/10^−9^ M) on *IL-1RI *((a), (b)), *IL-1RII* ((c), (d)), and *IL-6R*β** ((e), (f)) mRNA transcription in epithelial cells (*n* = 5) and stromal cells (*n* = 5) after 24 h incubation. Results are normalized against ACTB. Data are presented as fold induction relative to control. Asterisks indicate significant differences (*P < 0.05; **P < 0.01; ***P < 0.001) from the respective control, as determined by nonparametric one-way ANOVA *Kruskala*-*Wallisa* followed by *Dunns'a test*.

**Figure 7:**
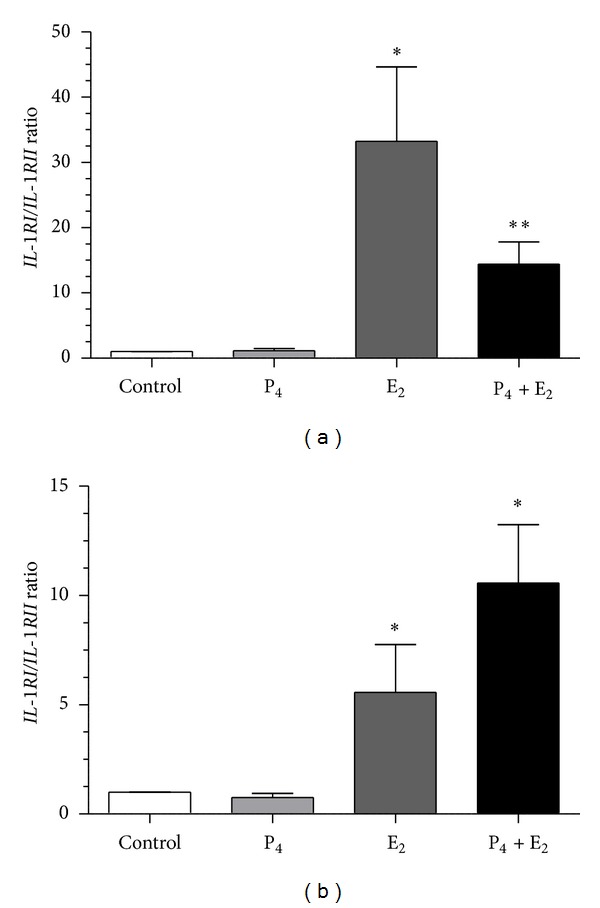
The effect of P_4_ (10^−7^ M), E_2_ (10^−9^ M), and P_4_ + E_2_ (10^−7^/10^−9^ M) on *IL-1RI:IL-1RII* mRNA transcription ratio in epithelial cells ((a); *n* = 5) and stromal cells ((b); *n* = 5) after 24 h incubation. Results are normalized against ACTB. Asterisks indicate significant differences (*P < 0.05; **P < 0.01) from the respective control, as determined by nonparametric one-way ANOVA *Kruskala*-*Wallisa* followed by *Dunns'a test*.

17-*β* estradiol and/or P_4_ upregulated *IL-1RI* mRNA transcription in epithelial cell compared to control group (*P* < 0.01; Figure 6(a)). 17-*β* estradiol or P_4_ upregulated *IL-1RII* mRNA transcription in epithelial cells compared to control group (*P* < 0.001; Figure 6(c)). In turn, E_2_ downregulated *IL-1RI* and P4 + E2 downregulated *IL-1RII* mRNA transcription in stromal cells compared to control group (*P* < 0.001; Figures 6(b) and 6(d)). 17-*β* estradiol and P_4_ + E_2_ upregulated *IL-6R*β** mRNA transcription in epithelial cells compared to control group (*P* < 0.01; Figure 6(e)). 17-*β* estradiol and/or P_4_ upregulated *IL-6R*β** mRNA transcription in stromal cells compared to control group (*P* < 0.05; Figure 6(f)). Additionally, E_2_ and P_4_ + E_2_ increased the *IL-1RI*/*IL-1RII* mRNA transcription ratio in epithelial and stromal cells (*P* < 0.05; Figures 7(a) and 7(b)).

## 4. Discussion

A short number of studies characterized IL in the equine endometrium [23, 30]. However, we have recently described IL-1*α*, IL-1*β*, and IL-6 immunolocalization in the equine endometrium [23]. We also showed that those ILs stimulated PG secretion through the upregulation of *PG synthases* mRNAs transcription in endometrial explants *in vitro* [23]. Additionally, the equine endometrial upregulation of *IL-1*α** and *IL-6* mRNAs transcription together with an upward tendency in *IL-1*β** mRNA transcription was demonstrated in early pregnancy [30]. However, it has been shown that ovarian steroids did not affect *IL-1*α** and *IL-6* mRNAs transcription in endometrial explants *in vitro* [30].

Interleukins stimulate PG production by endometrium in many species besides the mare [12, 31–34]. Tamura et al. [33] and Huang et al. [34] showed that IL-1*β* upregulated PGE2 secretion and PTGS-2 mRNA transcription in human stromal endometrial cells. Furthermore, the ability to produce PG in response to IL-1*α* in rat stromal cells was confirmed by Bany and Kennedy [31]. In turn, Chen et al. [32] confirmed that human epithelial cells are able to produce PG in response to IL-1*α* if the culture medium is supplemented with arachidonic acid (AA). Several previous studies present diverse conclusions [12, 31–35]. Tanikawa et al. [12] showed that IL1*α* and IL1*β* stimulated in a dose-dependent manner both PGE2 and PGF2*α* production in bovine stromal cells, but this stimulatory effect of both IL1s was not observed in epithelial cells. Nonetheless, Betts and Hansen [35] showed that IL-1*β* had no effect on PG production in bovine stromal cells, but augmented PGE2 and PGF2*α* production by epithelial cells. A comprehensive study concerning IL-6 influence on PG production by endometrial cells is lacking. Our previous data showed that IL-6 stimulated PG production by equine endometrial explants in vitro [23].

Cyclic changes in the endometrium are a complex process governed by the interplay of several signaling pathways that critically regulate cell growth and proliferation. Ovarian steroids play a key role in these processes. In the present study, we showed that ovarian steroids are not only triggering endometrial PG production, but they also modulate endometrial cell response to IL. As previously shown, ovarian steroids stimulated PG production by equine of epithelial and stromal cells [4]. The analysis of our present findings shows that E_2_ can be a modulator of endometrial cell response to IL. Additionally, it was found that the response to IL-1*α* and IL-6 could be strongly modulated, when compared to IL-1*β*.

It was shown that in the mare IL-1*α* and IL-6, expression is upregulated during the follicular phase of the estrous cycle, following E_2_ action [23]. It should be highlighted that the endometrial tissue response to acting factors results from epithelial and stromal cell activity. Our findings showed that the influence of ovarian steroids on IL-1*α*-stimulated PG production is dependent on the cell or PG types. Additionally, we showed that IL-6-stimulated PG production is strongly increased by E_2_. Interestingly, although we had seen no effect of IL-6 on PGE_2_ secretion by stromal cells, the pretreatment with E_2_ and P_4_ + E_2_ caused an increase of PGE_2_ secretion. In turn, the modulation of IL-1*β* influence on PG production by ovarian steroids is less evident, when compared to IL-1*α* or IL-6. However, it should be noted that IL-1*β* alone has the strongest influence on PG production from endometrial cells compared to IL-1*α* or IL-6. Regarding the present findings, the interaction between PG, IL, and ovarian steroids may be crucial for the local regulation of equine endometrium.

Baring in mind that endometrial IL-1*α* and IL-6 expression are upregulated in follicular phase of the estrous cycle, their promotion of endometrial cell proliferation and also that E_2_ enhanced their support of PG production; it may be assumed that these cytokines may play a role in local changes, such as angiogenesis, cell proliferation, and other processes taking place in endometrium. Additionally, the cross-talk between PG, IL, and ovarian steroids is highly likely to be determinant for implantation.

17-*β* estradiol positively affected IL-1*α*- and IL-6-stimulated PGE_2_ production may be a possible mechanism responsible for the angiogenesis regulation and cell proliferation in endometrium. Prostaglandin E_2_ acts in an auto-/paracrine manner on proangiogenic factors such as vascular endothelial growth factor (VEGF) secretion and angiopoietin-1 and angiopoietin-2 expression [36–38]. Additionally, PGE_2_ and PGF_2*α*_ influenced epithelial cells proliferation in human endometrial cells [7, 8]. Tsujii and DuBois [9] showed that PGE_2_ enhanced proliferation. It seems that IL-1*α* and IL-6 act on cell proliferation directly and indirectly by PG stimulation.

Once Haneda et al. [30] could not detect the link between ovarian steroids action and IL-1*α* and IL-6 expression around implantation period, it is possible that this interaction on that stage of the pregnancy is related to PG production. Synchronized development of the embryo to the blastocyst stage, endometrium differentiation to the receptive state, and cross-talk between the blastocyst and uterine luminal epithelium are fundamental to the implantation process [39]. In mice and rats, E_2_ is essential for preparation of the P_4_-primed uterus to the receptive state [39]. Various factors including cytokines, growth factors, homeobox transcription factors, and PG participate in embryo implantation through auto-/paracrine and/or juxtacrine mechanisms [39]. In contrast to other species, in the horse, the role of PG and IL on the embryo implantation is not described. It was demonstrated that the targeted disruption of *PTGS-2* is the cause of multiple failures in murine female reproductive processes that include ovulation, fertilization, implantation, and decidualization [40, 41]. The concentrations of PGs are elevated in the areas of increased endometrial vascular permeability associated with the initiation of implantation [42, 43] and exogenous PGs can reverse, at least partially, the effects of indomethacin on implantation in rat [44]. In turn, IL-1*α* and IL-1*β* are involved in production of leukemia inhibitory factor (LIF) [45], granulocyte-macrophage colony-stimulating factor (GM-CSF), and colony stimulating factor-1 (CSF-1) production [46]. Interleukin 1 stimulates production of metalloproteinases (MMP) and components of the plasminogen activator (PA)/PA-inhibitor cascade [47, 48] and also decreases connexin 43 in human endometrial stromal cells [49]. It has also been suggested that IL-6 may contribute to trophoblast growth and placental development in humans [50]. In our present work we pointed out the potential interaction between E_2_, IL, and PG. We suggest that E_2_ modulation of IL-1*α*- and IL-6-stimulated PG production during the preimplantation period may participate in event orchestration, including differentiation of endometrial cells and the vascular endothelial cell changes accompanying implantation and subsequent placental development. However, further studies are required to confirm these assumptions.

The pretreatment with combination of P_4_ and E_2_ augmented PG production in response to IL in endometrial cells. However, this enhancement, with one exception, was always followed by E_2_ increase of IL-stimulated PG production. Thus, one may conclude that E_2_ mainly increases IL-stimulated PG production. These results suggest that the mechanism responsible for enhancement of IL-stimulated PG production by steroids is different in epithelial and stromal cells. In epithelial cells, this mechanism is associated to *IL-1RI* mRNA transcription after ovarian steroids treatment, suggesting that ovarian steroids increase IL-1 effect on PG production via upregulation of IL-1RI expression. In stromal cells, a single treatment with E_2_ and P_4_ did not affect IL-1RI mRNA transcription. Possible mediators of E_2_ action in stromal cells are protein kinase A (PKA), nuclear factor-*κ*B (NF-*κ*B), and/or extracellular-signal-regulated kinases 1/2 (ERK1/2), which have been reported to be involved in the control of PGE2 secretion and PTGS-2 expression in response to IL-1*β* in human stromal cells [51] and the upregulation of PTGS-2, through PKA activation in different types of tissues [52, 53]. As an example, E_2_ has been shown to interact with NF*κ*B and to modulate its activity [54], stimulating PKA activity in hippocampal neurons [55, 56]. However, new studies are required to clarify the enhancement of IL-stimulated PG production by steroids. Additionally, our findings confirmed that E_2_ regulates IL-6-stimulated PG production through upregulation of *IL-6R*β** mRNA. However, the influence of E_2_ on IL-6R*α* expression should be investigated in the future.

In summary, it has been shown for the first time that E_2_ enhance IL-1*α* and IL-6-stimulated PG production. It may be one of the mechanisms responsible for local orchestrating events in endometrial tissue during estrous cycle and implantation. Additionally, we suggest that E_2_ influence on IL-1- and IL-6-stimulated PG production may result from activation of *IL-1RI* and *IL-6R*β** mRNAs transcription in endometrial cells.
